# Ultrafiltration of α-Lactalbumin Protein: Acquaintance of the Filtration Performance by Membrane Structure and Surface Alteration

**DOI:** 10.3390/polym13213632

**Published:** 2021-10-21

**Authors:** Nasrul Arahman, Cut Meurah Rosnelly, Yusni Yusni, Afrillia Fahrina, Silmina Silmina, Aulia Chintia Ambarita, Muhammad Roil Bilad, Poernomo Gunawan, Saeid Rajabzadeh, Ryosuke Takagi, Hideto Matsuyama, Muhammad Aziz

**Affiliations:** 1 Department of Chemical Engineering, Universitas Syiah Kuala, Banda Aceh 23111, Indonesia; cut.meurah@che.unsyiah.ac.id (C.M.R.); minaelsilmina@gmail.com (S.S.); 2 Magister Program of Environmental Management, Universitas Syiah Kuala, Banda Aceh 23111, Indonesia; 3 Research Center for Environmental and Natural Resources, Universitas Syiah Kuala, Jl. Hamzah Fansuri, No. 4, Darussalam, Banda Aceh 23111, Indonesia; 4 Doctoral Program, School of Engineering, Universitas Syiah Kuala, Jl. Syeh A. Rauf, No. 7, Banda Aceh 23111, Indonesia; afrilliafahrina26@gmail.com (A.F.); auliachinta@gmail.com (A.C.A.); 5 Department of Physiology, Universitas Syiah Kuala, Banda Aceh 23111, Indonesia; yusni@unsyiah.ac.id; 6 Faculty of Integrated Technologies, Universiti Brunei Darussalam, Bandar Seri Begawan BE1410, Brunei; roil.bilad@ubd.edu.bn; 7 School of Chemical & Biomedical Engineering, Nanyang Technological University, Singapore 627833, Singapore; pgunawan@ntu.edu.sg; 8 Research Center for Membrane and Film Technology, Department of Chemical Science and Engineering, Kobe University, Rokkodai-Cho 1-1, Nadaku, Kobe 657-0000, Japan; rajabzadehk@people.kobe-u.ac.jp (S.R.); takagi@harbor.kobe-u.ac.jp (R.T.); matuyama@kobe-u.ac.jp (H.M.); 9 Institute of Industrial Science, The University of Tokyo, 4-6-1 Komaba, Meguro-ku, Tokyo 153-8505, Japan; maziz@iis.u-tokyo.ac.jp

**Keywords:** polyethersulfone, α-lactalbumin, single-walled carbon nanotube, multi-walled carbon nanotube, membrane roughness

## Abstract

α-Lactalbumin is an essential protein with multiple roles in physiological and the nutritional functionalities, such as diabetic prevention, blood pressure stabilization, and cancer cell inhibition. In the present work, polyethersulfone (PES)-based membranes were developed by incorporating Pluronic F127 and carbon nanotubes with single- and multi-walled dimensions (Sw-Cnts and Mw-Cnts) as additives. The resulting membranes were evaluated for use in the filtration of α-lactalbumin protein solution. Four series of membranes, including PES pristine membrane, were fabricated via the phase inversion process. The characteristics of the membrane samples were analyzed in terms of morphology, membrane surface hydrophilicity and roughness, and surface chemistry. The characterization results show that the incorporation of additive increased the surface wettability by reducing the surface water contact angle from 80.4° to 64.1° by adding F127 and Mw-Cnt additives. The highest pure water permeability of 135 L/(m^2^·h·bar) was also exhibited by the PES/F127/Mw-Cnt membrane. The performance of the modified membranes was clearly better than the pristine PSF for α-lactalbumin solution filtration. The permeability of α-lactalbumin solution increased from 9.0 L/(m^2^·h·bar) for the pristine PES membrane to 10.5, 11.0 and 11.5 L/(m^2^·h·bar) for membranes loaded with Pluronic F127, Sw-Cnts, and Mw-Cnts, respectively. Those increments corresponded to 17, 22, and 28%. Such increments could be achieved without altering the α-lactalbumin rejections of 80%. Remarkably, the rejection for the membrane loaded with Sw-Cnts even increased to 89%.

## 1. Introduction

Bovine α-lactalbumin (B-α-La) is the second-largest protein contained in whey. It is an acidic and strong Ca^2+^-binding protein with four disulfide bonds, consisting of 123 amino acids in a single peptide chain [1,2]. Because it contains highly essential amino acids, α-lactalbumin (α-La) is rich in physiological and nutritional functionalities. It can stimulate the immune system and organize the production of lactose. In addition, it could hinder the increment of blood glucose and maintain the blood pressure of hypertensive patients. Furthermore, it could prevent the growth of some cancer cells [2,3,4]. Therefore, the isolation of B-α-La is desirable due to its excellent functionality and high commercial value.

Various techniques have been explored to isolate α-La from whey. They include chromatography, membrane separation, and isoelectric precipitation [5]. Among them, ultrafiltration (UF) is seen as highly attractive. Unlike the chromatography process, ultrafiltration does not require extensive salts, buffers, or chemicals and secures the product from any contamination. Furthermore, UF is easy to scale up and to run under continuous operation [6].

Membrane filtration is extensively explored for the recovery of medicinal substances from whey or soy protein by manipulating membrane material or operational conditions to achieve a high substance recovery and system productivity. Arunkumar and Etzel (2014) utilized UF with positively charged membrane material combined with tangential flow in a two-stage process to separate α-La and β-Lg based on isoelectric point (4.4 vs. 5.2, respectively). A positive charge was imposed on a 300 kDa cellulose membrane, while the solution pH and ionic strength were adjusted too, resulting in 87% of the pure α-La recovery [6]. Lucas et al. (1998) also reported high recovery of α-La from acid casein whey protein concentrate by employing positive charged inorganic membranes [7]. In another report, α-La purification from acid casein whey could be achieved by employing a series UF and diafiltration [8].

Moreover, a ceramic UF membrane was used to concentrate α-La in a continuous process to achieve a purity of 44% and recovery yield of 53% [9]. The fractionation of whey proteins was also achieved using UF membranes with different pore sizes and materials (polysulphone, polyethersulphone, polyamide, and cellulose) [10]. The cellulose membrane achieved the best selectivity for a-lactalbumin with minimal fouling. Despite extensive research on α-La fractionation and recovery, limited research is available on the application of polyethersulfone (PES) membrane, which became the focus of this study.

PES has been widely used in the industry, with applications such as fabrication of porous materials with higher porosity. PES is often used for membrane fabrication and has many advantages. It has a strong mechanical trait, is stable at high temperatures, and has resistance to corrosive chemical substances [11]. The porous PES structure is beneficial for enhanced permeability [12,13], which is desirable for whey protein filtration. PES is also soluble in several organic solvents, which makes it attractive to be converted into the membrane through the phase inversion process [14]. It can be custom-made for the recovery of α-La. Despite offering many advantages, PES polymers are hydrophobic, making pristine PES-based membranes prone to fouling and particularly detrimental when used to process whey. This limitation has been addressed in previous research through chemical modification and the blending of PES with hydrophilic polymers/nanoparticles [15,16] and many other approaches. There are a few common additives to enhance the hydrophilicity of PES-based membrane. They include polyethylene glycol, polyvinylpyrrolidone, Pluronic, tetronic [17,18,19], and many others. Recently, the use of inorganic particles as additives in the preparation of membranes has been reported. Dispersing inorganic particles in the dope solution improved the performance of the resulting membranes. They improved the hydrophilicity, imposed antifouling characteristics, and strengthened the mechanical traits of the resulting membranes [14].

This paper reports the development of a PES-based UF membrane tailored for α-La separation from whey protein by incorporating organic Pluronic and inorganic pore-forming agents. The inorganic pore-forming agents were single-walled carbon nanotubes (Sw-Cnts) and multi-walled carbon nanotubes (Mw-Cnts). The incorporation of those additives was performed to produce membranes with improved hydrophilicity and overall structure. Firstly, four membrane samples with different formulations were fabricated via the phase inversion process. The resulting membrane samples were then characterized to envisage the effect of the pore-forming agents on their structure and the surface chemistry. Finally, the membranes were assessed for the separation of B-α-La from whey protein.

## 2. Materials and Methods

### 2.1. Chemicals

The chemicals used in this study were PES Ultrason^®^ E 6020 P (BASF Co., Ludwigshafen, Germany), dimethylformamide (DMF) (Merck, Taufkirchen, Germany), Sw-Cnt (0.78 nm), and Mw-Cnt (7–15 nm × 0.5–10 µm (Sigma Aldrich, St. Louis, MI, USA). For a model of a pharmaceutical compound, α-La (Sigma Aldrich) was used in the feed. Other supporting chemicals for reagents and analysis were sodium dihydrogen phosphate dihydrate (NaH_2_PO_4_·H_2_O) and disodium hydrogen phosphate dehydrates (Na_2_H_2_PO_4_·H_2_O) (Merck, Darmstadt, Germany).

### 2.2. Membrane Fabrication

**Figure 1:**
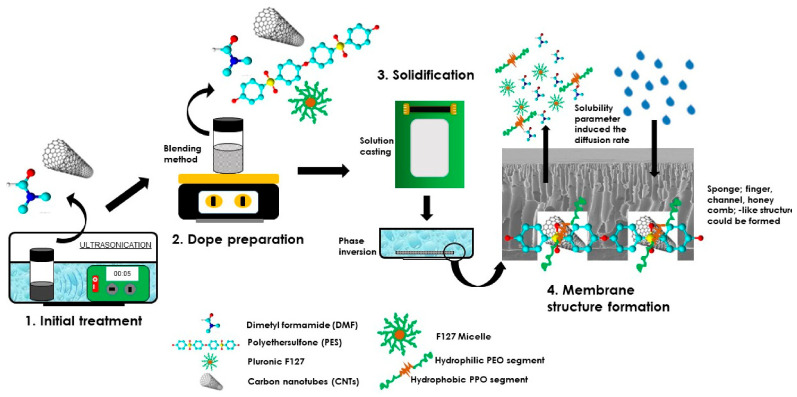
Illustration of membrane preparation using the phase inversion method.

Initially, a small amount (0.01 wt.%) of single carbon nanotubes (Sw-Cnts) and multi-walled carbon nanotubes (Mw-Cnts) were dispersed in DMF using an ultrasonic instrument in 5 min in a vial bottle. Then, PES (16 wt.%) was added to the solution, followed by Pluronic F-127 (3 wt.%). Detailed compositions of the prepared membrane solutions are listed in Table 1. After an overnight stirring, the dope solution was homogenous and ready to be used for membrane casting. It was cast on a flat squared-glass using a membrane applicator, followed by immersion into a bath containing pure water acting as the non-solvent (Figure 1).

### 2.3. Membrane Characterization

#### 2.3.1. Microstructure Investigation

The membrane morphology and surface roughness were evaluated using scanning electron microscopy (SEM, JSM-7500F, JEOL Ltd., Tokyo, Japan) and atomic force microscopy (AFM, SII NanoTechnology, Inc., Tokyo, Japan, SPA400), respectively. The cross-section specimen for SEM analysis was prepared by immersing the samples in liquid nitrogen, followed by freeze drying (Eyela, EDU-1200, Tokyo, Japan) at a temperature of –55 °C; then, they were fractured under those conditions to obtain a good cut. Then, all samples were sputtered using an osmium coater (Neoc-STB, Meiwafosis Co., Ltd., Tokyo, Japan). Finally, the surface and the cross-section morphological images were obtained at 5.00 and 2.00 kV. The samples for the surface roughness (Ra) were treated the same as the SEM samples, except they were not coated. After being placed on the AFM sample panel, the images were captured using a microcantilever (SI-DF40) support, with a scan area of 1 mm × 1 mm. The obtained data were further processed using Spicel32 software.

#### 2.3.2. Water Contact Angle (WCA)

Membrane surface wettability was evaluated by measuring the water contact angle using a goniometer (Drop Master 300, Kyowa Interface Science Co., Ltd., Tokyo, Japan, CA-A). A dried membrane sample was put on a glass panel; then, by using a microneedle, a 1 µL drop of water was dropped on the membrane surface. The angles between the water drop and the flat surface were automatically recorded on the monitor. The measurements of wettability were taken ten times, and they were presented as the average value and standard deviation.

#### 2.3.3. Chemical Groups

Fourier transform infrared (FTIR) spectroscopy (Thermo Scientific Nicolet iS5 FTIR with iD5 ATR, Thermo Fisher Scientific Inc., Waltham, MA, USA) was used to acquire the chemical groups of the pristine and the modified membranes. IR spectra of membranes were scanned at a wavenumber span of 400–4000 cm^−1^.

### 2.4. Membrane Filtration Performance

#### 2.4.1. Water Permeability

The water flux was analyzed to study the membrane filtration performance using a cross-flow filtration cell. A round sheet membrane coupon with an effective area of 9.075 cm^2^ was placed in the cell, and the filtration test was conducted under a constant transmembrane pressure of 1 bar. The volume of the water permeate that passed through the membrane was recorded at an interval of 10 min. The permeability data were recorded after the membrane was compacted for two hours. The water permeability was evaluated using Equation (1).

(1) $$\mathrm{Wp}=\frac{\mathrm{Vm}}{A.t.P},$$

where Wp is the water permeability (L/(m^2^·h·bar)), V is volume of permeate (L), A is membrane surface area (m^2^), t is time interval (h), and P is applied pressure (1 bar).

#### 2.4.2. Filtration of Lactalbumin Solution

The α-La solution (1 g/L) was prepared in a beaker glass (100 mL) by dissolving 100 mg of α-La into 100 mL of a homogeneous solution consisting of 50 mM NaCl and 1 mM Na_2_HPO_4_. Next, NaOH solution was added drop-wisely to the mixture to adjust the pH to 10.6, then stirred for a couple of hours until a homogeneous solution was obtained. 

The filtration performance was analyzed via filtration of the α-La solution using a cross-flow filtration cell. The protocol for the filtration test was similar to the water permeability test. After two hours of compaction, the water was replaced with the α-La solution as the feed. The permeate flux of the α-La solution was recorded every 10 min until the flux value was constant. The concentration of the α-La before and after filtration was analyzed using a spectrophotometer (Shimadzu Spectrophotometer UV-1800, Kyoto, Japan). The Wp of the α-La solution was evaluated using Equation (1), while the α-La rejection was calculated using Equation (2).

(2) $$R_{\alpha -\mathrm{La}}=\frac{C_{f}-C_{p}}{C_{p}}\times 100\%$$

## 3. Results and Discussion

### 3.1. Membrane Morphological Structure

**Figure 2:**
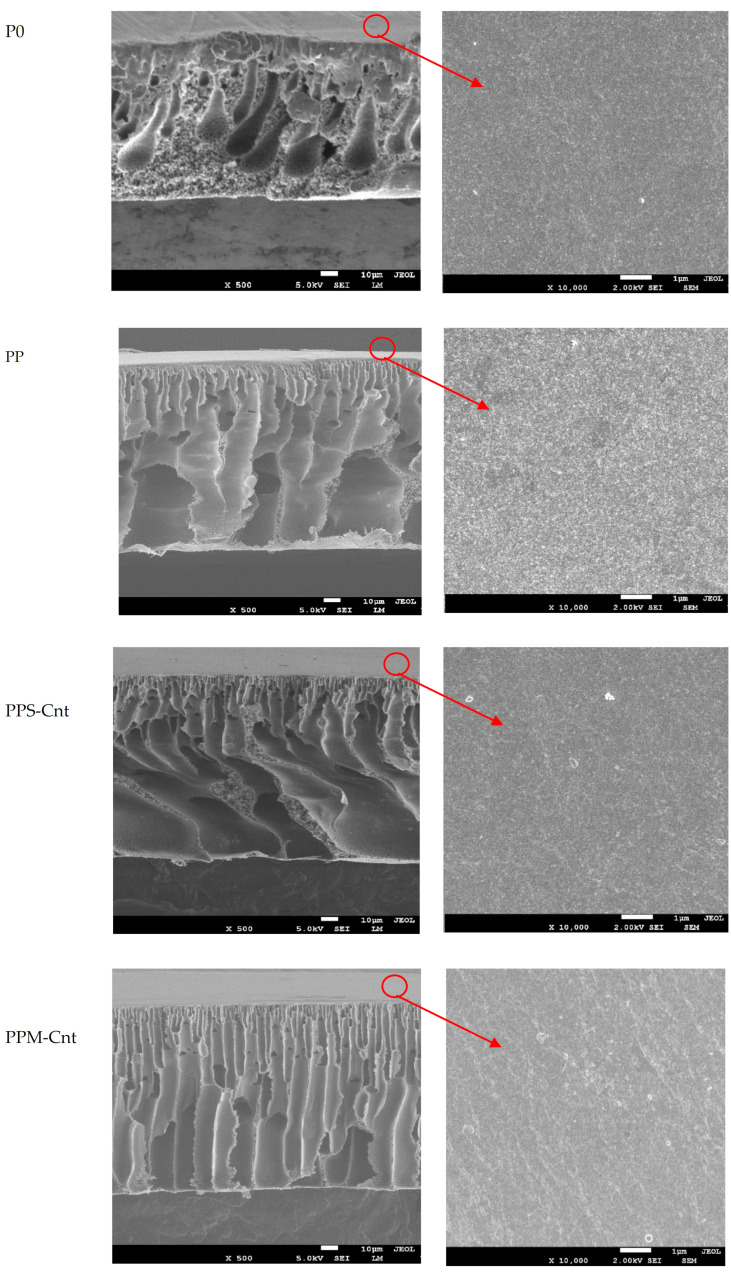
Membrane morphology analyzed by SEM.

Figure 2 shows that no morphological difference on the surfaces of all membranes. The cross-section images show that all membrane samples had an asymmetric structure that consisted of a dense top skin layer supported by layers with macrovoids. Nonetheless, the cross-section membranes depict the effect of additives loading towards the membrane structures. Loading of additives led to larger macrovoids (PP and PPS-Cnt) or a finger-like morphology (PPM-Cnt). 

The shapes of the cavities, such as macrovoid finger-like, sponge-like, and channel-like structures, are strongly related to kinetic aspects during the exchange of solvent and non-solvent in the coagulation bath [20,21,22]. The diffusion rate of the solvent and the non-solvent exchange act as a driving force determining the final morphological structure. The rate of solvent–non-solvent inter-diffusion depends on the value of solubility parameters of the solvent and non-solvent [23]. After the addition of Pluronic F-127 (in PP, PPS-Cnt and PPM-Cnt), the growth of finger-like voids increased. Pluronic F127 is an amphiphilic copolymer, generated by two substituents consisting of polyethylene oxide (PEO) (hydrophilic chain) and polypropylene oxide (PPO) (hydrophobic chain) [21,24]. Due to its hydrophobic nature, PPO tends to have a strong bond with the PES polymer chain.

Furthermore, the presence of the PEO segments in the exterior of micelle accelerated the in-diffusion rate into the casted polymer solution film. Consequently, when the Pluronic F127 micelles were attracted from the dope solution by the non-solvent, a more porous sub-layer was formed. The final voids determine the pore size of the membranes [21,24].

This study employed Cnts (single/multi-walled dimension) as dope solution additives to improve the resulting membrane characteristics. The presence of Sw-CNT and Mw-CNT particles promotes the formation of selective pores (channel-like void) in the cross-section membrane (see Figure 2). The introduction of nano-channels enhanced the resulting membranes’ water flux, selectivity, and mechanical properties [25,26]. Although the nano-carbons are naturally hydrophobic, the presence of Pluronic F-127 seemed to improve the hydrophilicity of Cnt and Mw-Cnt containing membranes—as later proved from the WCA results—through the hydrophobic bonding of PPO and carbon.

### 3.2. Membrane Surface Roughness

**Figure 3:**
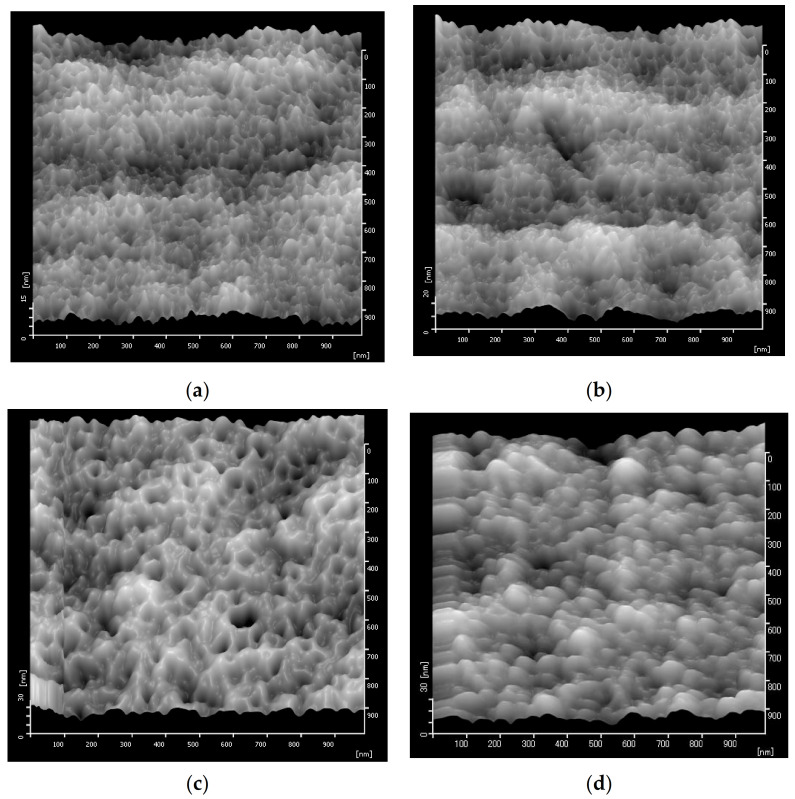
Membrane surface roughness analyzed by AFM: (**a**) P0, (**b**) PP, (**c**) PPS-Cnt, and (**d**) PPM-Cnt.

Figure 3 shows the 2D and 3D surface AFM images of the prepared membrane samples. The surface roughness parameters of all membranes are listed in Table 2. The photos show peaks with brighter colors and valleys with darker colors. The latter indicates the membrane pores. The PES pristine membrane (P0) had the smoothest surface with a Ra value of 2.04 nm. After the addition of an additive, the Ra values significantly increased (>6.04 nm). The presence of Pluronic F-127 in membrane solution led to the formation of more pores representing by higher surface roughness in Figure 3. Furthermore, the additions of Cnts and Mw-Cnts also led to enhancement of the pore formation, as shown by the slight increment of the Ra values to 6.23 nm and 6.85 nm, respectively.

### 3.3. Membrane Surface Wettability

**Figure 4:**
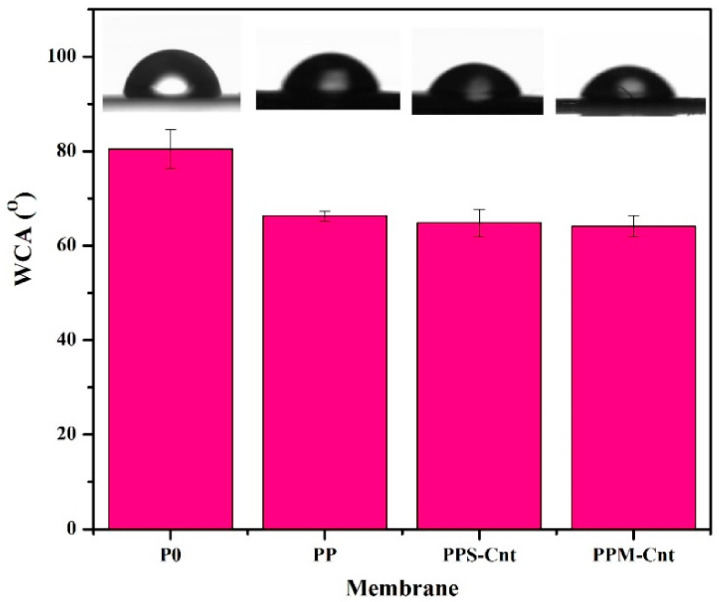
Membrane surface water contact angle (WCA).

The WCA was used to confirm the membrane surface wettability. A hydrophilic membrane has a contact angle <90°, while the hydrophobic one has a WCA >90°. Lower contact angle is associated with better wettability. Figure 4 shows that the pristine PES membrane (P0) has the highest WCA of 80.4°. After adding Pluronic F-127 in the PP membrane, the presence of ‒OH in PEO segments decreased the WCA to 66.3°, which is beneficial for membrane fouling alleviation when used for α-La filtration (Section 3.6). The hydrophilic surface imposes an antifouling attribute by generating a thin layer of water that prevents the adhesion of organic matters directly onto the membrane surface [27,28]. The presence of Cnts and Mw-Cnts in the PPS-Cnt and PPM-Cnt membranes tended to decrease the WCA slightly to 64.8° and 64.1° from the PP membrane (Figure 4).

### 3.4. Membrane Chemical Groups

**Figure 5:**
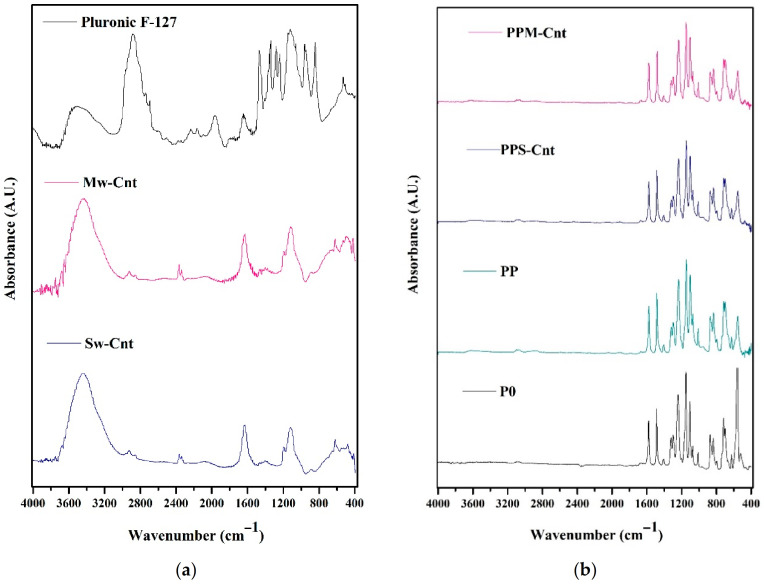
The ATR-FTIR spectra of additives (**a**) and all membranes (**b**) water droplet on top of the membranes’ surfaces.

Figure 5a presents the IR spectra of all additives employed to enhance membrane properties. The strong adsorption band intensity in the 2900 cm^−1^ in Pluronic spectra indicate the stretching vibrations of the C–H groups. The broad peak in wavenumber of 1100–1070 cm^−1^ expresses the presence of C-O in the Pluronic F127 [29]. Carbon nanotubes with single- and multi-walled dimensions show similar IR spectra. O–H groups are represented by the peaks around 3200–3400 cm^−1^, while the C–H stretch appears at ∼2920 cm^−1^. C = C stretching, C–O–H, and the C–C–C bending are indicated by the peaks around 1653, 1385, and 1090 cm^−1^, respectively [30].

Figure 5b shows FTIR spectra of P0, PP, PPS-Cnt, and PPM-Cnt membranes. P0 shows a strong C-O stretching band at 1106–1151 cm^−1^, strong S = O stretching (sulfoxide) and S=O stretching (sulfone) bands at 1072 cm^−1^, and 1322 cm^−1^, respectively [31]. A strong aromatic ester group C-O stretching band is shown at 1299 cm^−1^ [32]. The PP membrane shows a broader peak than P0 at a wavelength of 1450 cm^−1^ (assigned to the C-H in alkane group). It also has a C-O stretching band at 1077–1100 cm^−1^ [33]. The array of peaks between 1485–1575 cm^−1^ confirmed the broad peak of C-H bond, also appearing in P0 membrane. The C-H stretching band at 2840–3000 cm^−1^ of the pure Pluronic addictive (as shown in Figure 5a indicates that the C-H alkane group could have disappeared. The low peak at 3600 cm^−1^ present for all modified membranes is suggested to be due to O–H groups from the Pluronic F127 chain—the hydrogen bonding helps to enhance the surface hydrophilicity of membranes, which explains the WCA trend in Figure 4. Meanwhile, the PPS-Cnt has no significant change in FTIR spectra when compared to PPM-Cnt. Both PPS-Cnt and PPM-Cnt show similar peaks at wavelengths of 1588 (C=C, cyclic alkene) and 1070 cm^1^ (strong binding S=O stretching sulfoxide).

### 3.5. Membrane Permeability and Rejection Performance

**Figure 6:**
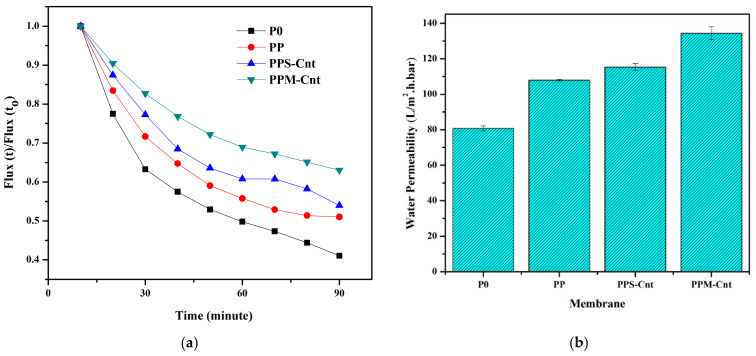
Membrane water permeability evaluated by the cross-flow system showing (**a**) evolution as function of time and (**b**) the steady state permeability.

Water permeability has a strong correlation with surface hydrophilicity and membrane porosity. Figure 6a,b are the same data source plotted in different figures, wherein Figure 6a shows the flux ratio with data uniformity for 90 min, while Figure 6b shows water permeability data when reaching a constant value. Overall, both show a tendency from the lowest rate to the highest flux ratio and permeability; they are P0, PP, PPS-Cnt, and PPM-Cnt, respectively. Specifically, Figure 6a presents the increase in membrane water flux ratio for the membranes prepared from dope solutions containing additives (Pluronic and inorganics). The pristine PES membrane (P0) initially posed the lowest water permeability (Figure 6b), while after loading with an additive, the water permeability was enhanced by up to 40% from 82 L/(m^2^·h·bar) to 135 L/(m^2^·h·bar) for PPM-Cnt. Loadings of Pluronic F-127 and carbon nanotubes likely promoted the formation of more pores (higher Ra in Figure 2). The cross-sectional structure of the PPM-Cnt membrane provides a uniform finger-like channel about 10 nm in diameter. The formation of a large number of pores facilitated a higher water path to pass through the membrane than the P0 with a lack of voids. Therefore, the PPM-Cnt exhibits the highest water permeability. Furthermore, hydrogen bonding promotes higher hydrophilicity in the membrane surface, which attracts more water, resulting in higher water permeation.

### 3.6. Membrane Performance in α-La Solution Filtration

**Figure 7:**
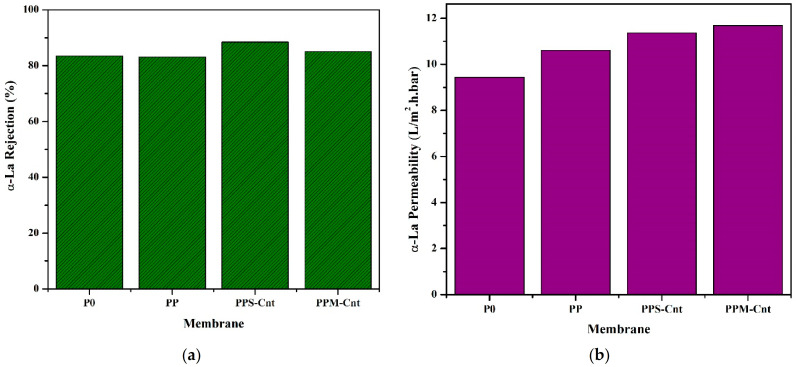
Filtration performance of the lactalbumin solution: (**a**) rejection and (**b**) permeability.

Figure 7 presents the permeability of α-La solution, showing that the addition of Pluronic F127, Sw-Cnts, and Mw-Cnts increased the membrane permeation compared to the pristine membrane (P0). All membranes show the same tendency in α-La flux as the pure water permeation in Figure 6. However, the value of α-La permeability decreases significantly compared to the pure water permeability. For instance, the highest performance employed by PPM-Cnts showed that flux permeation cut down from 135 L/(m^2^·h·bar) (water permeability) to 11.3 L/(m^2^·h·bar) (α-La permeability). It dropped significantly, with a permeability loss of up to 91% due to fouling from α-La compound in the feed solution. Some membrane properties influenced the filtration performance, including hydrophilicity, roughness, porosity, and charge. Those properties dictate the interaction between the molecules in the feed and membrane surface [34,35].

The rejection performances of all membranes towards α-La molecules were almost similar. PPS-Cnt membrane, which consists of F127 and Cnt, had the highest rejection of 88.5%, while the neat PES membrane (P0) had the rejection value of 83.46%. The loading of F127 in PES membrane slightly decreased the rejection to 83.11% (PP membrane) due to more significant porosity in the membrane surface and, possibly, pore enlargement [36,37]. PPM-Cnt membrane consisting of F127 and Mw-Cnt showed α-La rejection rate of 85.07%. The enhancement of α-La rejection can be attributed to higher hydrophilicity in the membrane surface. Overall, the addition of carbon nanotubes (in PPS-Cnts and PPM-Cnts) showed better permeation and rejection values than the pristine membrane.

Figure 7 clearly shows that the performance of the modified membranes was better than the pristine PSF. The permeability of α-lactalbumin solution increased from 9 L/(m^2^·h·bar) for P0 membrane to 10.5, 11.0 and 11.5 L/(m^2^·h·bar) for PP, PPS-Cnt, and PPS-Mnt membranes, respectively. Those increments corresponded to 17, 22, and 28%. Such increments were achieved without altering the rejections of 80%. Remarkably, the rejection for PPS-Cnt even increased to 89%. The hydrophilic surface attracts more water to pass through the membrane and the porous structure in the cross-section membrane facilitates higher water permeate flux. In rejection performance, the hydrophilic surface prevents the organic molecules with hydrophobic nature from attaching to the membrane surface. Then, channel-like structures in cross-section membranes with smaller sizes (10 nm) retain organic molecules that pass through the membrane. Therefore, the presence of Sw-CNTs and Mw-CNTs increase both permeability and selectivity.

## 4. Conclusions

This study reports the development of PES-based membranes for α-La filtration. It was carried out by the incorporation of Pluronic F127, Sw-Cnts, and Mw-Cnts as pore-forming additives. The incorporation of those additives was proven to improve the properties and the filterability of the resulting membranes. Dosing the additive in the dope solution led to the formation of a membrane with higher porosity and surface pores with finger-like cross-sectional morphology. The membrane also imposed hydrophilicity by lowering the WCA from 80° to 60°. Those changes in surface chemistry and structure were proved to be effective in lowering the intrinsic membrane resistance up to 40% relative to the pristine PES membrane. After incorporating additives, the membranes could retain up to 89% of α-La and offered a higher flux of up to 28%. The overall findings emphasize the potential of PES/F127/S-Cnt membrane α-La recovery from whey protein. The findings in this work can be used as a good starting point for further development of PSF-based membrane by incorporating Cnt additives.

## Figures and Tables

**Table 1:** Composition of dope solution for membrane fabrication.

Membrane		Concentration			
	PES	Pluronic F-127	Sw-Cnt	Mw-Cnt	DMF
P0	16	0	0	0	84
PP	16	3	0	0	81
PPS-Cnt	16	3	0.01	0	80.99
PPM-Cnt	16	3	0	0.01	80.99

**Table 2:** Roughness parameter of the ultimate membranes.

Membrane	Ra (nm)	RMS (nm)
P0	2.04	2.54
PP	6.04	7.51
PPS-Cnt	6.23	8.69
PPM-Cnt	6.85	8.85

## Data Availability

Available on request to the corresponding author.
